# Ascorbic Acid Priming Boosts Cotton Seed Chilling Tolerance via Membrane Stability and Antioxidant Cycles

**DOI:** 10.3390/plants14203122

**Published:** 2025-10-10

**Authors:** Peng Han, Haixia Ma, Lu Lu, Jincheng Zhu, Xinhui Nie, Jianwei Xu, Zhibo Li

**Affiliations:** Key Laboratory of Oasis Ecology Agricultural of Xinjiang Bingtuan, Agricultural College, Shihezi University, Shihezi 832003, China

**Keywords:** cotton, low temperature stress, ascorbic acid priming, regulation mechanism, antioxidant systems

## Abstract

Low-temperature stress severely restricts cotton seed germination and seedling establishment, especially in early spring. Ascorbic acid (AsA) priming is a promising strategy to enhance stress tolerance, yet its mechanisms in cotton remain unclear. This study examined the effects of AsA priming on seed germination at 15 °C. Seeds were treated with 0, 25, 50, or 100 mg/L AsA for 3, 6, 12, or 24 h. Results showed that 50 mg/L AsA for 24 h significantly improved germination potential, rate, index, and promptness index (*p* < 0.05). Compared with water-primed seeds, AsA-primed seeds exhibited greater radicle length (+17.67%) and fresh weight (+136.26%) under chilling stress. This treatment markedly increased antioxidant enzyme activities, including POD (+196.74%), SOD (+43.81%), and CAT (+49.43%), while also promoting the accumulation of Ascorbate–Glutathione cycle-related enzymes and metabolites, thereby reinforcing the antioxidant defense system. Multidimensional statistical analyses further indicated that AsA enhanced root growth by stimulating antioxidant defenses while inducing a trade-off that slightly reduced fresh weight, suggesting a balance between growth and oxidative protection. Overall, AsA priming improves cotton seed cold tolerance by activating enzymatic and non-enzymatic antioxidant systems and mediating a growth–defense trade-off, underscoring its potential as an effective priming agent for early sowing under low-temperature stress.

## 1. Introduction

Owing to its unique ecological conditions, Xinjiang has emerged as China’s largest cotton production region, accounting for approximately 92.2% of the nation’s total cotton output in 2024 [1]. Cotton (*Gossypium hirsutum* L.) exhibits particular sensitivity to temperature fluctuations during its growth stages, with emerging cotyledons demonstrating heightened vulnerability to low-temperature stress during germination phases [2,3]. This physiological response renders young seedlings susceptible to cold stress damage, significantly compromising germination rates [4,5]. Late-spring cold events, characterized by abrupt temperature drops after initial spring warming [6,7], frequently occur in the region. These events can cause frost damage, growth delays, and reductions in both yield and fiber quality [2,5,8].

Cold stress significantly impacts cotton germination and growth, particularly affecting its physiological and biochemical processes [5,9]. Low temperatures inhibit the electron transport chains in mitochondria and chloroplasts, leading to increased electron leakage and a burst-like accumulation of reactive oxygen species (ROS) [10], such as superoxide anions (O_2_^−^) and hydrogen peroxide (H_2_O_2_) [11]. Concurrently, the activities of antioxidant enzymes, including superoxide dismutase (SOD), peroxidase (POD), and catalase (CAT), are suppressed under low-temperature conditions, disrupting the balance between ROS production and scavenging [12,13,14]. As ROS accumulates, the degree of lipid peroxidation in cotton cell membranes intensifies, resulting in elevated malondialdehyde (MDA) levels, reduced membrane stability, and increased damage to cellular structures caused by cold stress [15,16]. This leads to ion leakage and impaired physiological functions, exacerbating physiological damage to the seeds [9,17]. In cotton production, in addition to selecting cold-resistant cotton varieties, exogenous hormones such as melatonin [18,19], salicylic acid [20,21], or nano-selenium [22,23] at appropriate concentrations are utilized for seed soaking and germination promotion to mitigate the adverse effects of low temperatures on seed germination, thereby improving germination rates and seedling survival rates.

Ascorbic acid (AsA), a fundamental antioxidant synthesized in plants, plays essential roles in plant growth, development, and adaptation to environmental stresses [24,25,26]. In the ascorbate–glutathione (AsA-GSH) cycle, AsA reduces H_2_O_2_ to water via ascorbate peroxidase (APX), becoming oxidized to monodehydroascorbate (MDHA) and oxidized ascorbic acid (DHA) [27,28]. These oxidized forms are subsequently recycled back to active AsA by interactions with GSH and NADPH, ensuring continuous ROS detoxification and maintaining cellular redox homeostasis [29]. By stabilizing cell membranes and protecting metabolic enzymes, AsA preserves the integrity of cellular structures and supports normal physiological processes such as seed germination [25] and seedling growth [30]. Beyond its direct antioxidant role, AsA promotes the accumulation of proline and other osmoprotectants [31], elevates chlorophyll content, and modulates secondary metabolism, together forming integrated protective mechanisms that mitigate the adverse impacts of stress on plant growth [32]. Moreover, AsA influences stress adaptation at the transcriptional level, as it regulates the expression of key biosynthetic and signaling genes, including GDP-L-galactose phosphorylase in the L-galactose pathway [33] and stress-responsive transcription factors such as ANAC019, ANAC072, and ZAT10, thereby linking its metabolic functions with gene regulatory networks [34,35]. This dual function, as both an antioxidant and a regulator of gene expression, positions AsA as a key factor in developing cold tolerance in plants.

This study aims to investigate the effects of AsA seed priming on the germination characteristics and physiological responses of cotton variety Xinluzao 61 under low-temperature conditions. The specific objectives are (i) to evaluate the effects of different AsA concentrations on seed germination rate and speed; (ii) to analyze the regulation of antioxidant enzyme activities and the glutathione cycle in seeds by AsA priming; and (iii) to elucidate the physiological mechanisms by which AsA priming enhances low-temperature tolerance in cotton seeds. These results will provide a theoretical basis for understanding and managing cotton seed germination under cold stress, while offering practical strategies to mitigate the negative effects of late-spring cold spells on early-sown cotton. These insights contribute to the development of sustainable cotton production in regions prone to sudden low-temperature events.

## 2. Result

### 2.1. Effects of AsA Priming with Different Concentrations and Times on Cotton Seed Germination

**Figure 1:**
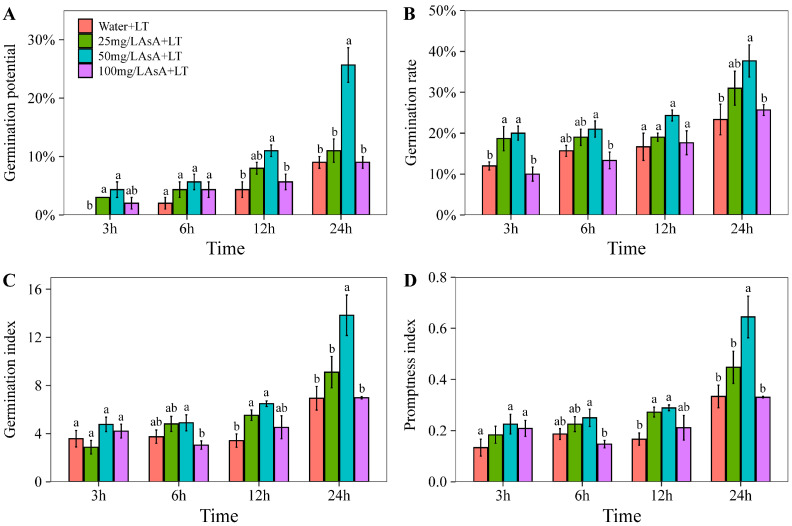
Effects of AsA priming at different concentrations and times on cotton seed germination. Note: The effects of AsA priming on germination potentials (**A**), germination rates (**B**), germination index (**C**), and promptness index (**D**). Values are means ± SD of five biological replicates (*n* = 5). Different lowercase letters indicate statistically significant differences among treatments at *p* < 0.05 according to one-way ANOVA followed by Duncan’s multiple range test.

Under low-temperature conditions, the germination and seedling growth of cotton seeds are significantly affected. Priming with AsA has demonstrated positive effects on the germination potential (Figure 1A, GP), germination rate (Figure 1B, GR), germination index (Figure 1C, GI), and promptness index (Figure 1D, PI) of cotton seeds. Improvements in these indices were significant (*p* < 0.05) with extended priming durations. Compared to the control group (soaking in water only, W+LT), the AsA-primed groups exhibited higher values for GP, GR, GI, and PI, indicating that AsA priming significantly influences (*p* < 0.05) cotton seed germination and stress resistance across various concentrations and durations. Notably, during the 24 h priming durations, enhancements in GP (11.11% to 25.56%), GR (31.11% to 37.78%), GI (9.10 to 13.84), and PI (0.45 to 0.64) were most significant (*p* < 0.05) when the AsA concentration increased from 25 mg/L to 50 mg/L, but improvements significantly (*p* < 0.05) reduced with further increases to 100 mg/L. In particular, a 50 mg/L AsA concentration with a 24 h priming period resulted in the highest values for GP (25.56% ± 4.16%), GR (37.78% ± 5.67%), GI (13.84 ± 2.38), and PI (0.64 ± 0.12) among all priming conditions. These findings suggest that this combination of 50 mg/L AsA and a 24 h priming duration is an effective method for enhancing the cold stress tolerance of cotton seeds.

### 2.2. Effects of Appropriate AsA Priming on Seed Growth Under Low-Temperature (15 °C) Stress

**Figure 2:**
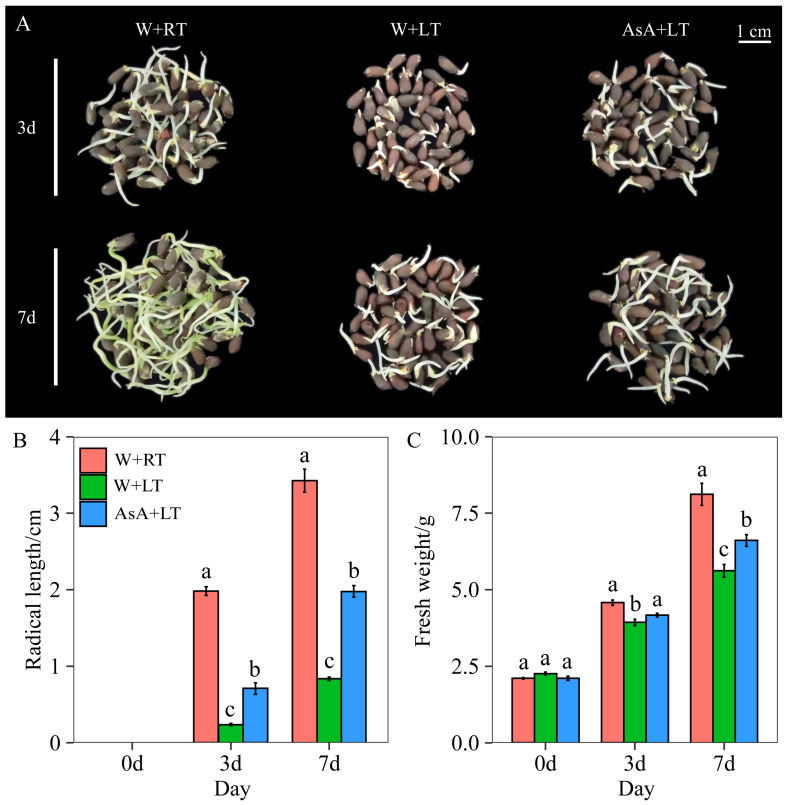
Effects of different germination temperatures and priming treatments on seed germination. Note: The effects of different priming treatments at varying germination temperatures on seed germination status (**A**), radicle length (**B**), and fresh weight (**C**). Values are means ± SD of five biological replicates (*n* = 5). Different lowercase letters indicate statistically significant differences among treatments at *p* < 0.05 according to one-way ANOVA followed by Duncan’s multiple range test.

According to the above results, three distinct treatments for cotton seed germination were conducted to evaluate the effects of AsA priming and temperature conditions on seed vigor and germinative performance. The treatments were designed as follows: (i) W+RT (seeds soaked in distilled water for 24 h and continuously germinated at a root temperature of 25 °C); (ii) AsA+LT (seeds primed with the optimal AsA priming conditions for 24 h and then germinated at a low temperature of 15 °C), and (iii) W+LT (seeds soaked in distilled water for 24 h at room temperature and then germinated at a low temperature of 15 °C). Large differences were observed among the cotton seeds in various stages of development, from germination to the establishment of roots (Figure 2A). By the third day of seed germination, the performance of seeds germinated at 25 °C was indeed significantly (*p* < 0.05) superior to that of seeds germinated at 15 °C. Specifically, both the radicle length (RL) and the fresh weight (FW) of seeds germinated at 25 °C were substantially greater than those of seeds germinated at 15 °C. Under the germination conditions at 15 °C, the AsA+LT treatment demonstrated a markedly higher enhancement in seed growth indicators compared to the W+LT treatment. Specifically, on the 3rd day of germination, the FW and RL increased by 6.00% and 200.00%, respectively, in the AsA+LT treatment when compared to the W+LT treatment. By the 7th day of germination, these enhancements were even more pronounced, with increases of 17.67% for FW and an impressive 136.26% for RL. Therefore, the beneficial effects of AsA priming on cotton seed germination are especially evident under low-temperature conditions.

### 2.3. Impact of Appropriate AsA Priming on Cell Membrane Stability, and Antioxidant Enzyme Activities Under Low-Temperature (15 °C) Stress

**Figure 3:**
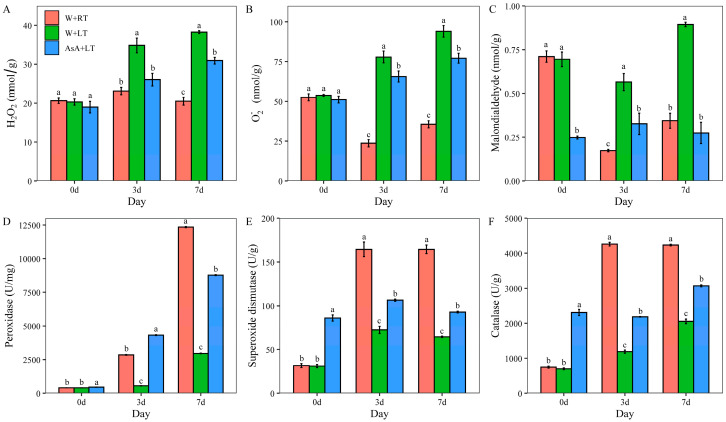
Effects of different germination temperatures and priming treatments on cell membrane stability and antioxidant enzyme activities of cotton seeds. Note: The effects of different priming treatments at varying germination temperatures on the content of H_2_O_2_ (**A**), O_2_^−^ (**B**), MDA (**C**), POD (**D**), SOD (**E**), and CAT (**F**), respectively. Values are means ± SD of five biological replicates (*n* = 5). Different lowercase letters indicate statistically significant differences at *p* < 0.05 according to one-way ANOVA followed by Duncan’s multiple range test.

Low-temperature stress triggers excessive ROS, including H_2_O_2_ and O_2_^−^, in plants, causing cellular damage, including DNA, protein, and membrane destruction, along with lipid peroxidation and MDA accumulation, which compromises cell stability. Under W+RT treatment, ROS (H_2_O_2_ and O_2_^−^) and MDA levels in cotton seeds remained stable, indicating balanced oxidative metabolism. In contrast, W+LT treatments significantly (*p* < 0.05) disrupted this balance, sharply increasing H_2_O_2_, O_2_^−^, and MDA levels by 67.63%, 190.02%, and 180.76%, respectively (Figure 3A–C), leading to severe oxidative damage during germination. Exogenous AsA pretreatment effectively alleviated these adverse effects: compared to the W+LT group, AsA treatment reduced H_2_O_2_ and O_2_^−^ levels by 22.10% (Figure 3A) and 16.94% (Figure 3B), respectively, and significantly (*p* < 0.05) inhibited lipid peroxidation, lowering MDA accumulation by 58.90% (Figure 3C). Simultaneously, AsA priming enhanced antioxidant enzyme activities, including POD, SOD, and CAT. Under room temperature, activities increased by 10.9%, 176.56%, and 209.29%, respectively, before germination (Figure 3D–F). Under low-temperature stress, AsA further elevated POD, SOD, and CAT activities by 686.16%, 46.99%, and 84.04% on day 3, with POD showing the most substantial rise, and maintained higher levels through day 7 (196.74%, 43.81%, and 49.43%, respectively). These findings suggest that AsA enhances antioxidant defense, blocks ROS chain reactions, and protects cell membrane integrity, thereby improving cotton seed germination under low-temperature stress.

### 2.4. Effects of Suitable AsA Priming on Enzyme Activities and Metabolite Content in the Ascorbate-Glutathione Cycle Under Low-Temperature (15 °C) Stress

**Figure 4:**
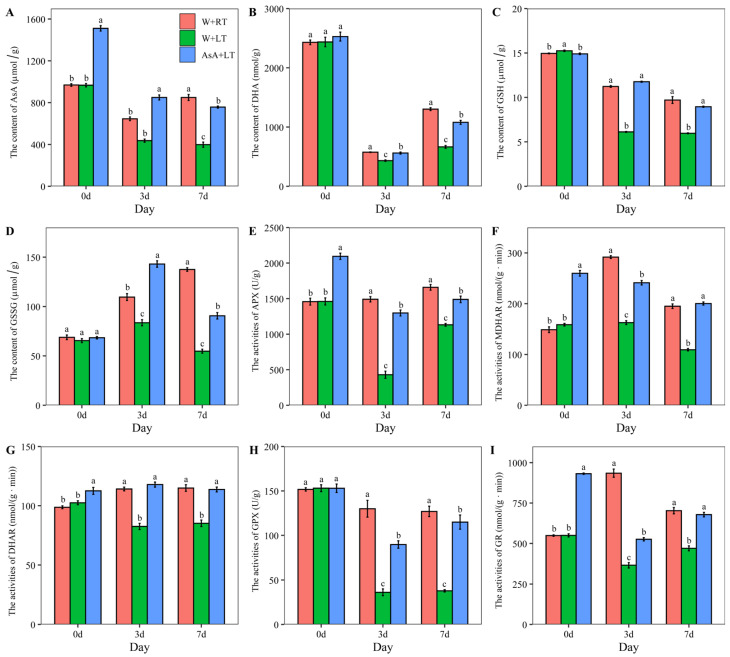
Effects of the suitable AsA priming on the contents of metabolites and enzyme activities in the AsA-GSH cycle. Note: The effects of AsA priming on the contents of AsA (**A**), DHA (**B**), GSH (**C**), and GSSG (**D**), and the enzyme activities of APX (**E**), MDHAR (**F**), DHAR (**G**), GPX (**H**), and GR (**I**). Values are means ± SD of five biological replicates (*n* = 5). Different lowercase letters indicate statistically significant differences at *p* < 0.05 according to one-way ANOVA followed by Duncan’s multiple range test.

The AsA-GSH cycle plays a crucial role in maintaining plant health and enhancing resistance to abiotic stress. As illustrated in Figure 4, most metabolites associated with the AsA-GSH cycle remained unaffected by AsA priming pretreatment, with the notable exception of reduced AsA, which showed significant (*p* < 0.05) alterations. In contrast, the activities of all enzymatic components except glutathione peroxidase (GPX) were markedly influenced by AsA priming prior to cotton seed germination under ambient temperature conditions. Comparative analysis revealed substantial declines in both enzyme activities and metabolite levels of the AsA-GSH cycle during W+LT relative to W+RT. However, AsA priming pretreatment (AsA+LT) effectively alleviated these stress-induced reductions, establishing statistically significant (*p* < 0.05) differences between AsA+LT and W+LT groups. Remarkably, certain enzymatic activities and metabolite concentrations in low-temperature germinated seeds were restored to levels comparable to those observed under normal germination conditions. By day 7 of germination, no significant differences (*p* > 0.05) existed between AsA+LT and W+RT groups in the activities of MDHAR, DHAR, and GR, nor in the concentrations of reduced GSH. Notably, the mitigation efficacy of AsA priming displayed temporal dynamics under prolonged low-temperature stress. The enhancement rates of most enzymatic activities and metabolite levels in AsA+LT seeds progressively diminished compared to W+LT controls as germination progressed. At day 3 post-treatment, the percentage increases in enzymatic activities were quantified as follows: APX (202.17%), MDHAR (48.27%), DHAR (42.79%), GPX (147.69%), and GR (43.94%). By day 7, these enhancement rates shifted to APX (31.64%), MDHAR (83.22%), DHAR (33.51%), GPX (203.82%), and GR (44.45%). Parallel trends were observed in metabolite levels, with day 3 increases in AsA (94.60%), DHA (29.62%), GSH (92.15%), and GSSG (71.20%), transitioning to day 7 values of AsA (90.23%), DHA (62.23%), GSH (50.13%), and GSSG (65.50%).

### 2.5. Synergistic Effects and Trade-Off Mechanisms of Antioxidant Metabolism in Cold-Resistant Germination

**Figure 5:**
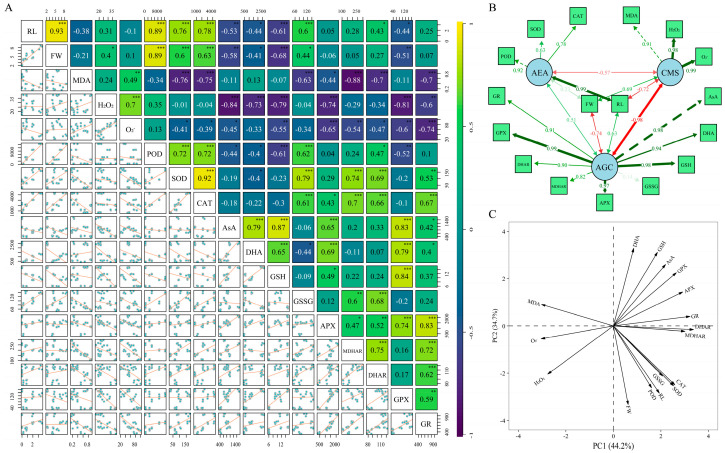
Analysis of the interrelationships among cell membrane stability, antioxidant enzyme activity, and the AsA-GSH cycle during cold-resistant germination of cotton seeds. Note: (**A**) Correlation matrix showing the pairwise relationships among seedling traits (RL, radicle length; FW, fresh weight), cell membrane stability indicators (MDA, H_2_O_2_, O_2_^−^), antioxidant enzyme activities (POD, SOD, CAT), metabolites in the AsA-GSH cycle (AsA, DHA, GSH, GSSG), and the related enzyme activities (APX, MDHAR, DHAR, GPX, GR). * means that *p* ≤ 0.05, ** means that *p* ≤ 0.01, and *** means that *p* ≤ 0.001. (**B**) Structural equation modeling (SEM) illustrating the direct and indirect effects of antioxidant enzyme activity (AEA), cell membrane stability (CMS), and the AsA-GSH cycle (AGC) on cold-resistant germination. Green arrows represent positive effects, red arrows represent negative effects, and line thickness indicates effect strength. Numbers beside arrows represent standardized path coefficients; arrow thickness indicates effect strength; solid arrows denote significant paths (*p* < 0.05 or *p* < 0.01), whereas dashed arrows denote less significant or nonsignificant paths (*p* ≥ 0.05). (**C**) Principal component analysis (PCA) showing the distribution and contributions of different physiological and biochemical parameters to germination variation.

To comprehensively understand the effect of AsA on the low-temperature resistance of cotton seeds, we employed Spearman correlation analysis, structural equation modeling (SEM), and principal component analysis (PCA) to systematically examine cell membrane stability (CMS), antioxidant enzyme activity (AEA), and the role of the AsA-GSH cycle in the anti-cold germination process. PCA was used to reduce dimensionality and identify dominant patterns among antioxidant and stress-related variables, while SEM quantified the direct and indirect effects of these factors on seed growth. Our analysis further elucidates the interrelationships between these factors in seed development. Spearman correlation analysis (Figure 5A) revealed that CMS-indicators (MDA, H_2_O_2_, and O_2_^−^) were negatively correlated with root length (RL) (r = −0.38 to −0.10) but had little effect on fresh weight (FW). In contrast, AEA-indicators (POD, SOD, and CAT) significantly promoted RL (r > 0.75, *p* < 0.01). However, antioxidant compounds within AGC, such as AsA, GSH, and APX, exhibited an inhibitory effect on FW (r < −0.50), suggesting that high antioxidant accumulation may compete with biomass growth under low-temperature stress. These correlations provided a basis for further SEM analysis to explore the relative contributions of CMS, AEA, and AGC to seed growth. SEM analysis (Figure 5B) provided further insights into these relationships. CMS significantly inhibited RL (Std.all = −0.716), reflecting oxidative damage to cellular structures. Conversely, AEA had a strong positive effect on RL (Std.all = 0.991), highlighting its key role in promoting root growth under low-temperature stress. AGC displayed a dual effect: it enhanced RL (Std.all = 0.626) but reduced FW, indicating a potential resource trade-off within antioxidant metabolism. These SEM results indicate that antioxidant metabolism promotes root development but may compete with overall biomass accumulation, reflecting resource allocation trade-offs under stress conditions. PCA results (Figure 5C) supported these observations. PC1 was primarily associated with antioxidant enzymes (APX, GPX, and GR) and antioxidant molecules (AsA, GSH), representing overall antioxidant metabolic activity. CMS-related indicators also loaded strongly on PC1, reflecting the intensity of oxidative stress. PC1 explained 44.2% of the total variance, underscoring its dominant role. PC2 captured the trade-off between growth and antioxidant metabolism: DHA, GSH, and AsA exhibited higher positive loadings, while RL, FW, POD, SOD, and CAT exhibited strong negative loadings. This pattern indicates that investment in antioxidant metabolism may come at the cost of growth potential under stress conditions. In conclusion, CMS contributes to cellular stability by inhibiting root growth, AEA plays a critical role in RL promotion, and AGC negatively impacts FW, highlighting the competitive dynamics of antioxidant cycles in energy metabolism. PCA confirms the complex interplay among oxidative stress, antioxidant systems, and seed growth, suggesting that plants regulate antioxidant metabolism and resource allocation to balance growth and survival under stress conditions.

## 3. Discussion

### 3.1. AsA Priming to Enhance Cotton Seed Germination at Low Temperatures

Improving the germination ability of cotton seeds under low-temperature conditions is of great significance for enhancing early spring sowing survival and yield [4]. The results of this study demonstrated that AsA priming significantly promoted cotton seed germination, particularly under low-temperature stress. This finding not only enriches our understanding of the physiological adaptation mechanisms of cotton seeds to low-temperature stress but also highlights the specific role of AsA in cotton breeding and production. Compared with traditional treatments [36,37,38,39,40], AsA priming is simple, efficient, and environmentally friendly, making it more suitable for practical application [41]. Especially in regions like Xinjiang, where late-spring cold snaps frequently occur, priming seeds with 50 mg/L AsA for 24 h can provide effective low-temperature protection, reduce early sowing failure, and improve field emergence, thereby ensuring early growth and final yield. Moreover, AsA priming is easy to implement within existing sowing practices without additional equipment, demonstrating good feasibility. Taken together, AsA priming is not only effective under laboratory conditions but also has clear practical application value, offering a feasible and eco-friendly strategy for early spring cotton cultivation and contributing to yield stability in regions prone to late-spring cold events.

### 3.2. AsA Priming Boosts Cotton Seed Germination Under Low-Temperature Stress by Enhancing Antioxidant Defense

AsA priming, as a natural antioxidant treatment, protects cells from oxidative damage, regulates key physiological processes, and enhances stress tolerance, thereby promoting plant growth and productivity [42,43]. Antioxidant regulatory networks display considerable diversity across different crops. In earlier investigations, rice and maize mainly depend on the coordinated action of APX and CAT to remove hydrogen peroxide [44,45], whereas Arabidopsis maintains ROS homeostasis through the peroxiredoxin/thioredoxin pathway [46]. In wheat and tomato, the accumulation of proline and secondary metabolites plays a key role in alleviating oxidative stress [47,48]. Together, these results reveal that crops have developed diverse and complementary defense systems to cope with oxidative challenges. This study demonstrated that AsA priming significantly enhanced both enzymatic and non-enzymatic antioxidant systems. In the enzymatic system, the activities of SOD, POD, CAT, and GPX were markedly increased, with GPX showing the greatest enhancement, indicating that cotton relies more heavily on GPX for ROS scavenging. Previous studies have also confirmed that the GPX gene family not only participates in oxidative stress defense but also regulates fiber development and stress tolerance [49]. In the non-enzymatic system, the levels of AsA, DHA, GSH, and GSSG were significantly elevated, synergistically maintaining intracellular redox homeostasis. Notably, cotton may depend more on the GSH-GSSG cycle to maintain redox balance under low-temperature conditions, thereby reducing oxidative damage.

Since cotton seeds are sensitive to low temperature and have a short germination period, AsA priming plays a particularly critical role during this developmental stage by enhancing antioxidant capacity. Future studies integrating transcriptomic analysis could systematically unravel the gene regulatory networks involved in AsA-mediated cold tolerance, identify key genes and signaling pathways, and thereby clarify the species-specific mechanisms of AsA priming, ultimately providing theoretical guidance for optimizing seed priming strategies under low-temperature stress.

### 3.3. Unraveling the Complex Interactions Among Oxidative Stress, Antioxidant Defense, and Seed Development

Recent studies have revealed an inevitable metabolic trade-off in resource allocation between defense and growth, where pathways involving ROS signaling [50], glucosinolates [51], and tryptophan-derived secondary metabolites [52] enhance resistance and adaptability but often reduce biomass accumulation, highlighting the universal growth cost of defense activation. Plants under cold stress need to fine-tune oxidative metabolism and resource allocation to maintain a delicate balance between growth and antioxidant defense [53,54,55]. While elevated antioxidant levels can mitigate oxidative damage, an overly robust antioxidant response may compromise plant growth and survival [56,57,58]. AsA priming, by regulating oxidative metabolism and the AsA-GSH cycle, reflects a cotton-specific physiological trade-off in which enhanced cellular protection may come at the expense of biomass accumulation. This “antioxidant investment” can be viewed as a survival-first strategy. This mechanism highlights the dual role of AsA in cold tolerance—not only as an antioxidant mitigating oxidative damage, but also as a regulator of resource allocation that determines the balance between “defense” and “growth.” For cotton, this trade-off is particularly critical, as the germination and early seedling stages are extremely vulnerable to chilling, and any imbalance in resource partitioning can directly affect stand establishment. At the applied level, combining AsA priming with agronomic practices such as nutrient-supplemented irrigation at emergence or fertilizer-coated seed treatments could help alleviate the resource allocation pressure, thereby sustaining growth while maintaining antioxidant protection. Moreover, co-application with other priming agents (salicylic acid or nitric oxide donors) may further enhance stress responses and reduce conflicts between growth and defense. A deeper understanding and targeted regulation of this physiological trade-off between antioxidant investment and growth provides both a theoretical foundation and practical pathway for improving cold tolerance and yield stability in cotton during early developmental stages.

## 4. Materials and Methods

### 4.1. Plant Materials

The test material is the cotton variety Xinluzao61, a primary cultivar well-suited for early maturing cotton regions and extensively cultivated in northern Xinjiang. The seeds were provided by the Xinjiang Academy of Agricultural Reclamation. Healthy and plump cotton seeds were sterilized by soaking in 75% alcohol for 5 min. Subsequently, the seeds were rinsed 4–5 times with sterilized distilled water to remove any residual liquid. Unless otherwise noted, all chemicals and solvents were of analytical grade and used as provided by the manufacturers.

### 4.2. Experimental Design

Experiment 1: To explore the effects of AsA on enhancing the cold tolerance of cotton seeds, sterilized seeds were primed with AsA at concentrations of 0, 25, 50, and 100 mg/L for 3, 6, 12, and 24 h in the dark at room temperature (25 °C). Each treatment was conducted in five independent biological replicates, with each replicate consisting of 60 seeds. The optimal AsA priming concentration and duration were determined by recording the main seed germination indicators after an 8-day period.

Experiment 2: Following the initial experiment to determine the optimal AsA priming conditions, we conducted a subsequent experiment to elucidate the antioxidative regulation mechanism by which AsA priming enhances the chilling tolerance of cotton seeds. After selecting and sterilizing the seeds, they were subjected to three distinct treatments: W+RT (seeds soaked in distilled water and continuously germinated at a root temperature of 25 °C), AsA+LT (seeds primed with the optimal AsA priming conditions and then germinated at a low temperature of 15 °C), and W+LT (seeds soaked in distilled water at room temperature and then germinated at a low temperature of 15 °C). Each treatment was conducted in five independent biological replicates, with each replicate consisting of 60 seeds. Seed growth indicators, the cell membrane stability index, and the main enzyme activities from the antioxidant system, as well as the relative enzyme and substance content of the AsA-GSH cycle, were analyzed on the 0th, 3rd, and 7th days of seed germination, respectively.

### 4.3. Seed Cultivation

After priming, seeds were rinsed with distilled water, gently dried, and air-dried to their original moisture content. Primed seeds were evenly placed in sterilized Petri dishes with moist filter paper and germinated in a light incubator at 15 °C. A 12 h light/dark cycle was applied, and moisture was maintained throughout the germination period.

### 4.4. Determination of Relevant Indicators of Seed Germination

Seed germination was recorded daily. Germination is defined as the point when the radicle length breaks through the seed coat and reaches half the length of the seed itself. Several indices were calculated to assess the cotton seeds’ germination performance [59]:

Germination Potential (GP): This was calculated as the percentage of seeds that had germinated by the third day, relative to the total number of experimental seeds, according to Formula (1):

(1) $$GP(\%)=\left(\frac{Number\;of\;seeds\;germinated\;on\;day\;3}{Total\;number\;of\;experimental\;seeds}\right)\times 100$$

Germination Rate (GR): This was determined as the percentage of seeds that had germinated by the eighth day, relative to the total number of experimental seeds, according to Formula (2):

(2) $$GR(\%)=\left(\frac{Number\;of\;seeds\;germinated\;on\;day\;8}{Total\;number\;of\;experimental\;seeds}\right)\times 100$$

Germination Index (GI): This was calculated as the sum of the daily germination rates, according to Formula (3):

(3) $$Gv=\sum \left(\frac{Gt}{Dt}\right)$$

where Dt is the day of the germination test, and Gt is the number of seeds germinated on that day.

Promptness Index (PI): This was calculated based on the germination rates on the first four days, according to Formula (4):

(4) PI=1.0×nd1+0.75×nd2+0.50×nd3+0.25×nd4

### 4.5. Determination of the Seed Growth Indicators

Before germination, and on the 3rd and 7th days after germination, cotton seeds from the W+RT, W+LT, and AsA+LT treatments were selected to measure the main seed growth indicators. The fresh weight (FW) was first recorded using a scale. After measuring FW, the samples were packed into Kraft paper bags, marked, and then dried. They were first dried at 105 °C for 30 min and then at 80 °C for 8 h until weight stability was achieved. The dry weight (DW) was then measured. The radicle length (RL) was measured using a vernier caliper.

### 4.6. Determination of the Enzyme Activities of the Antioxidant System

The activities of SOD, POD, and CAT were assessed to evaluate the antioxidant response in cotton seeds under various treatments. SOD activity, which is pivotal in neutralizing superoxide radicals by converting them into oxygen and H_2_O_2_, was measured using the nitro blue tetrazolium (NBT) method [60]. This method is based on the inhibition of NBT photoreduction by SOD, with the extent of inhibition correlating to SOD activity and the extent of inhibition was quantified by monitoring changes in optical density (OD) at 560 nm using a spectrophotometer.

The activity of POD, an enzyme that plays a role in the breakdown of H_2_O_2_ and other peroxides, indicating the general peroxidative capacity of the seeds, was determined using the guaiacol method [61]. In this assay, guaiacol is oxidized to tetra guaiacol in the presence of H_2_O_2_, producing a colored product, and the reaction progress was monitored spectrophotometrically by recording the increase in absorbance in the visible light range.

CAT activity, essential for the detoxification of H_2_O_2_ by catalyzing its decomposition into water and oxygen, was quantified by monitoring UV absorption at 240 nm [62]. The rate of decrease in absorbance over time, due to the breakdown of H_2_O_2_, is indicative of CAT activity.

### 4.7. Determination of MDA and Reactive Oxygen Species Contents in Seeds

The MDA content, an indicator of lipid peroxidation, was assessed using the thiobarbituric acid colorimetric method as described in reference [63]. For the determination of H_2_O_2_ levels, frozen seeds (0.5 g) were homogenized in 5 mL of pre-cooled acetone, followed by centrifugation at 12,000× *g* for 20 min at 4 °C. The supernatant was then used for the H_2_O_2_ content assay, which was performed according to the instructions provided with the assay kit (BC3595, Beijing Solarbio Science & Technology Co., Ltd., Beijing, China).

To visualize the superoxide anion (O_2_^−^) production, nitro-blue tetrazolium (NBT) staining was conducted following the protocol outlined by Daudi and O’Brien [64]. For the measurement of H_2_O_2_ in pistils, 10 mg of frozen tissue was homogenized in 3 mL of 10 mM 3-amino-1,2,4-triazole, centrifuged at 6000 rpm for 25 min at 4 °C, and the supernatant was reacted with 0.1% titanium tetrachloride in 20% H_2_SO_4_. The absorbance of the reaction mixture was measured at 410 nm, as detailed by Brennan and Frenkel [65].

### 4.8. Determination of the Enzyme’s Activities and Substance Content of Ascorbate-Glutathione Cycle

Enzyme activities of the AsA-GSH cycle, including APX, glutathione peroxidase (GPX), monodehydroascorbate reductase (MDHAR), dehydroascorbate reductase (DHAR), and mitochondrial glutathione reductase (GR), were assayed using specific commercial kits (Nanjing Jiancheng Bioengineering Institute, Nanjing, China). Concentrations of reduced AsA, DHA, reduced GSH, and oxidized glutathione (GSSG) were determined with the corresponding metabolite assay kits (Solarbio Science and Technology, Beijing, China).

### 4.9. Data Processing and Statistical Analysis

The data were sorted, calculated, and statistically analyzed using Microsoft Excel 2010 and the SPSS Statistics (v20.0) data processing system. Charts and figures were drawn using R Studio (v 2025.09.1+401). In this paper, all data are presented as the mean ± standard error (SE). The difference test was conducted using Duncan’s new multiple range test at a significance level of *p* ≤ 0.05. To reveal the interaction and the antioxidant effects that improve cotton seed chilling tolerance, critical indicators during low-temperature germination after AsA priming were analyzed using Correlation Analysis, Principal Component Analysis, stepwise Regression Analysis, and Path Analysis. A high degree of correlation indicates that the index is more important and plays a key role in promoting seed germination under low-temperature conditions.

## 5. Conclusions

In summary, seed priming with 50 mg/L AsA for 24 h significantly enhances cotton seed germination and cold tolerance. This effect is primarily mediated through the regulation of oxidative metabolism and the AsA-GSH cycle, which alleviates oxidative damage caused by low-temperature stress while maintaining a dynamic balance between antioxidant defense and growth. Future studies integrating transcriptomic and multi-omics approaches could further elucidate the regulatory effects of AsA on cold-responsive transcription factors and their downstream genes, assess its long-term impacts on subsequent growth, yield, and fiber quality, and perform expression and functional validation of key genes. These investigations will provide theoretical guidance and practical insights for breeding cold-tolerant cotton and optimizing management strategies to mitigate the effects of late-spring cold events, thereby enhancing yield stability.

## Data Availability

Data are contained within the article.
